# Economics of Dentition

**Published:** 1898-02

**Authors:** J. J. Madden


ARTICLE II.

### ECONOMICS OF DENTITION.

BY DR. J. J. MADDEN, '95.

Read before the Alumni Association, December 7th, 1897.

As young practitioners in dentistry, most of us desire to direct our energies in a course that will naturally have a tendency to build up a practice depending upon the training we have received during our college term, and the many expensive lessons tnat are taught us through our own failures. In all these trials, we are naturally weeding out the little egotism that had its birth when we received our diplomas, so that, not having drunk too deeply of the Fountain of Knowledge at our command, we may hear the warning word to keep on. Education never ends.

It is with some timidity tonight, I assure you, that I select this subject, which is no doubt familiar to all of you, and I shall attempt to point out in some parts of the paper some conditions in the course of dentition in which Nature shows her wonderful wisdom, and the economical way she has in bringing certain of these organs into action just where they are needed, and the exquisite and thoughtful manner in which she discards those not intended to serve throughout life.

The economy that Nature displays in her beautiful construction and arrangement of the teeth, is something that has attracted the attention of histologists and embryologist, causing them to neglect the study of other organs and admire the uniqueness of Nature's work in building up the teeth. She sets about her task as early as the sixth week of embryonic life, when the jaws are in the first stages of formation, and the lower jaw made up only of its supporting cartilaginous scaffold. The first sign seen in the development of the teeth would be a sort of a depression extending along the alveolar ridge, and a heaping up of cells on its surface. We now see the formation of the epithelian cord. This is of itself interesting, as the enamel organ is developed from it.

It will, I trust, be unnecessary for me to go into detail as to the changes that occur in the shape of this organ, only to say that as the dentine papilla develops about the fifteenth week, it is seen to push its way upward and change the enamel organ from a bud shape to a cup shaped formation, and in this way the developing tooth gradually takes on its certain form. And while the process is going on here, there is still another going on, namely, the growing up of the follicular wall and the union of its parts, cutting off the forming tooth from the epithelial cord and allowing it to live and grow in a comfortable little house that we know as the Dental Follicle. This process takes place with all the temporary teeth.

The first economical work we see is when, before or about the time Nature has finished her work with the part of the temporary teeth, she makes the epithelial cord pass down and plant the enamel germ of a permanent tooth behind and beneath the temporary tooth, and in this way each temporary tooth has its succeeding tooth beneath it, and in process of development.

Later on, we find the permanent teeth that do not succeed the other teeth are developed in a like manner. The first, or permanent molar receives its germ from the mucous membrane, the second and third molars being supplied in turn from the cord of the preceeding tooth.

The greater part of this work is done during embryonic life, and we must not lose sight of the fact that during this time the jaws are scarcely formed, and the lower jaw developing what we know as Meckels Cartilage; it is just beginning to receive its lime salts, and the bone cells are rapidly building up and around this cartilage, from the symphysis to the angle.

Leaving the developing teeth and jaws, I hurry on through fœtal life until birth, and it is here we find the laws of selection and economy obeyed as the little one comes into the world toothless. And why is this so? For the reason that teeth are not demanded, as Nature has fitted the mother to be the nutritional organism; she has designed to supply her with organs whose lacteal secretion is shown to be the best food for the infant.

I have often thought, why does not Nature begin with the eruption of the teeth at birth? But I find that the digestive tract and glands are not fully organized, and could not tolerate food that would require teeth to masticate it, so once more obeying the law, that which is best for the organism is tolerated.

We may pass on a few months, and when the teeth are required no one knows more about it than the baby, who intuitively attempts to bite its fingers, or to translate its meaning, it wants a rubber ring. It is now about five or six months old, and one day finds its mother happy beyond measure, and the father, after his delight, begins to think of sleepless nights and the dental bills of the future.

We find that the process of the first stage of dentition goes on from about the fifth to the thirtieth month, when all the temporary teeth are in position.

It is during this period that the child sometimes suffers considerably, and I am going to term it a *critical period in life*. I have never met with this term, and justify myself in using it because at this early period the infant is practically made up of a nervous system, the brain being proportionally larger, and the glands in the same condition, so that any deviation from the normal process may cause any condition, from that of fretfulness to those of convulsions or paroxysms, and this we can appreciate when we think of the teeth with open foramens, so that any restriction, such as the alveolus or dense gum tissue, will cause pressure on the sensitive and vascular underlying pulps.

Nature shows good judgment again, in that these teeth are not of dense structure, nor so delicately constructed as the permanent teeth; but suffice it to say, they are sufficient for the work.

While the temporary teeth are in service, Nature begins to introduce teeth that shall serve us during life, and now in the eruption of the first permanent teeth she again shows her wonderful workings in this way, that she demands none of the temporary teeth, but places at the ends of the temporary series the first permanent, or sixth year molars. In this result I believe I can see a threefold cause: First, that the jaws may be properly supported while she is at work in the anterior part of the mouth; second, that the young subject may have a better grinding surface while she is replacing other teeth; third, that it may be a strong barrier in keeping the posterior developing teeth in position. The last view seems to be supported by the fact that the first molars are the largest of the series.

We find now, through a physiological process of a somewhat obscure nature, that certain cells have been working assiduously in tearing down and carrying away the roots of the temporary teeth, and in this process we recognize the fact that it is not of a mechanical nature, due to pressure of the permanent teeth.

The cells doing this work may be called osteoclasts, cementoclasts, odontoclasts, with the presence of a certain enlarged papilla near the root of the temporary tooth.

The essential factor in this process will be a healthy temporary tooth, the presence of the cells referred to, and the papilla-like formation at the end of the root.

The jaws have all the time been expanding in all directions, so that when the permanent central incisor is ready to erupt it has sufficient space made, of course in a great measure depending upon the space left by the temporary tooth. The next teeth to be replaced are lateral incisors, and then the temporary molars, these substituted by permanent bicuspids. Now again we find that Nature has waited so that she may drive a wedge that shall form a keystone and supporting pillar to the arch, and she uses the root to form a prominence bearing the same name, which gives contour to the face, and is an important factor in expression.

The last molars or wisdom teeth are interesting to study, for we see here they have become almost rudimentary organs, and in many mouths this condition really exists, while in others the teeth fail to erupt.

This is due in a great part to the fact that the jaws will hardly accommodate them, as it is a retrograde process, not only in the jaws but also in the teeth.

You will observe that I have not attempted to give you any tables, nor do I want it felt that the subject was selected because we are unfamiliar with it. I trust it will be a factor in making all of us observers, that we may be enabled to get the most out of the subject that is before us.—*Dental Practitioner and Advertiser*.

